# Medical students perceptions of spiritual care

**DOI:** 10.3389/fmed.2026.1802023

**Published:** 2026-05-12

**Authors:** Gita Sekar Prihanti, Fahreza Hadi Firmansyah

**Affiliations:** 1 Department of Medical Education, Faculty of Medicine, University of Muhammadiyah Malang, Malang, Indonesia; 2 Department of Islamic Medicine, Faculty of Medicine, University of Muhammadiyah Malang, Malang, Indonesia

**Keywords:** spiritual care, Spirituality and Spiritual Care Rating Scale (SSCRS), cultural (Muslim) adaptation, medical students, medical and health education

## Abstract

**Introduction:**

This study aimed to assess medical students’ perceptions of spiritual care using a culturally adapted Muslim-context version of the Spirituality and Spiritual Care Rating Scale (SSCRS).

**Methods:**

A cross-sectional study was conducted among 470 medical students at the Faculty of Medicine, Universitas Muhammadiyah Malang. Participants were selected and data were collected through a self-administered questionnaire. Statistical analyses included independent *t*-tests, one-way ANOVA, Pearson’s correlation, and multivariate linear regression.

**Results:**

The mean total spiritual care perception score was 76.11 (SD = 7.02; range 54–90), indicating generally positive perceptions. Female students had significantly higher scores than males (76.59 ± 7.02; *p* = 0.032; CI (95%) −2.82; −0.13). A weak positive correlation was found between age and perception scores (*r* = 0.153, *p* = 0.001). Academic year was significantly associated with perception scores (*p* = 0.005), with clinical-stage students showing the highest mean scores (77.36 ± 7.17; CI (95%) 0.75;5.87). However, in multivariate analysis, only gender remained a significant predictor (*p* = 0.035).

**Discussion:**

These findings suggest that demographic factors, particularly gender, are associated with variations in medical students’ perceptions of spiritual care. The results highlight the importance of integrating spiritual care education into medical curricula. The generalizability of these findings is limited due to the single-institution design. However, the comprehensiveness of the analyses and the multiple characteristics (academic years and clinical stages) of the respondents within large sample size in this study provide a basis for further use of this result.

## 1 Introduction

The concept of holistic health views human beings as an integrated whole, encompassing physical, psychological, social, and spiritual dimensions that are interconnected and mutually influential (1). From an Islamic perspective, spiritual care is defined as the ability of healthcare professionals to understand patients’ uniqueness in accordance with their fitrah and inherent human nature (2). Nevertheless, many patients still perceive that their spiritual care needs have not been optimally fulfilled. This condition is attributed to healthcare professionals’ limited knowledge of and training in spiritual care (3).

Spiritual care is inherently relational and involves communication-based practices such as active listening, empathy, emotional support, and meaningful interaction with patients (4). The provision of spiritual care has been shown to yield various benefits for patients. Empirically, spirituality and religiosity are associated with better psychological adjustment, reduced anxiety and hopelessness, and greater resilience in the context of chronic illness (5). Therapeutic communication is essential in recognizing and responding to patients’ spiritual needs, as it promotes trust, facilitates patient-centered care, and strengthens healthcare providers’ competence in delivering spiritual care (6). Therefore, physicians, nurses, and other healthcare professionals are expected to possess the competence to deliver spiritual care as an integral component of a holistic health approach (7).

Spiritual care competencies of medical and health professionals grew as well as their professional identity and personal identity. Demographic factors as part of their personal identity, are characteristics inherent to individuals that distinguish one individual from another, such as age, gender, and academic year. An individual’s level of spirituality is inseparable from the influence of these demographic factors (8). Age differences, in particular, may influence a person’s perspective on life. A study conducted by Nurhayati et al. (9) demonstrated an association between age and an individual’s ability to find spiritual meaning when experiencing illness.

To date, studies examining medical students’ perceptions of spiritual care remain limited. Therefore, based on the issues outlined above, this study aims to analyze the perceptions of spiritual care among medical students. This study used a culturally adapted version of the Spirituality and Spiritual Care Rating Scale (SSCRS) for a Muslim context and was conducted at the Faculty of Medicine, Universitas Muhammadiyah Malang.

## 2 Literature review

Spirituality is abstract and subjective in nature and is associated with multiple assumptions. It may be understood as a relationship with God, nature, other people, and one’s surroundings (10). Spiritual care constitutes an essential component of holistic health and aims to fulfill patients’ spiritual needs. Spiritual care can be defined as care that allows humanity to reach others through presence, deep listening, empathy, and compassion (11). Although most nurses report having no formal training in providing spiritual care, nearly all indicate that they have cared for patients with spiritual needs (12).

The provision of spiritual care has been associated with several patient benefits, including improved quality of life, reduced anxiety, and the provision of hope. Patients whose spiritual needs have been met report higher levels of satisfaction with the healthcare services they receive (13).

Islamic spiritual care represents a form of spiritual care delivered based on Islamic religious values derived from the Qur’an and hadith. These are the primary sources of authority in Islam. The Qur’an is regarded as the literal word of God revealed to the Prophet Muhammad, while the Hadith consists of recorded sayings and actions of the Prophet that guide the interpretation and practice of Islamic teachings. Islamic spiritual care can be defined as the ability of healthcare providers to understand patients’ uniqueness in accordance with their inherent human nature (2). Spiritual care is not exclusively associated with palliative care. There are also forms of Islamic spiritual care such as muraqabah meditation, a meditation technique grounded in Islamic religious values. This meditation technique has been shown to assist patients experiencing depression and anxiety (14).

Perceptions of spirituality and spiritual care are influenced by several factors, including religion, cultural background, educational level, and work experience. Differences in age may give rise to variations in perspectives. Several studies have also reported that work setting and area of specialization influence differences in perceptions of spirituality and spiritual care (15).

## 3 Materials and methods

### 3.1 Research design

A cross-sectional research design was conducted at the Faculty of Medicine, Universitas Muhammadiyah Malang. The study was conducted in December 2022.

### 3.2 Participants

The population of this study consisted of students of the Faculty of Medicine, Universitas Muhammadiyah Malang. The study included 470 medical students from all academic years and clinical rotations. Sample size determined by logistic regression formula which consist of three independent variables and 96.7% as the proportion of high spiritual care perception. The total student population was 750, representing a response rate of 62.67%. Inclusion criteria were active Muslim students who consented to participate. Non-Muslim students and those unwilling to participate were excluded.

All participants provided informed consent prior to participation. Participants were contacted through institutional communication channels by medical educators or student representatives. After gaining access to the medical students, the survey was distributed using a Google Form. Informed consent was incorporated into the form. Students completed the questionnaire individually, and independence of responses was ensured by preventing discussion during the completion. Each participant completed the questionnaire only once. These procedures were implemented to ensure the authenticity and confidentiality of responses. Ethical approval was obtained from Ethics Committee at the Faculty of Medicine, Universitas Muhammadiyah Malang (No. E.5.a/0106/KEPK-UMM/IX/2022), with all data anonymized prior to analysis to protect respondent confidentiality.

### 3.3 Instrument

Data collection in this study employed a questionnaire using a culturally adapted version of the Spirituality and Spiritual Care Rating Scale (SSCRS) for a Muslim context. This questionnaire comprises five domains: existential element, religiosity, personalized care, spiritual care, and Muslim’s value.

The first version of the Spirituality and Spiritual Care Rating Scale (SSCRS) was developed by McSherry et al. (16) to assess nurses’ perceptions of spirituality and spiritual care in the United Kingdom. In its original version, the SSCRS questionnaire included domains that did not solely focus on spiritual care but also encompassed aspects of spirituality, spiritual care, religiosity, and personalized care. These four domains were represented by 17 items and were assessed using a Likert scale.

The questionnaire was subsequently translated into Indonesian by Mulyono and Chen (17) and modified in accordance with Islamic religious culture, resulting in the SSCRS cultural (Muslim) adaptation version. This questionnaire consists of 18 items divided into five domains: existential element, religiosity, personalized care, spiritual care, and Muslim’s value. The questionnaire has a Cronbach’s alpha coefficient of 0.64 and already valid. Compared with other instruments used to assess perceptions of spiritual care, the strength of this questionnaire lies in its adaptation to Islamic religious culture.

### 3.4 Statistical analysis

Data collected from respondents through the questionnaire were subsequently analyzed using IBM SPSS (Statistical Product and Service Solutions) version 25. For the analysis of the age variable, Pearson’s correlation test (sig. < 0.05) was applied. For the gender variable, an independent *t*-test was conducted, with a significance level of sig. < 0.05 indicating differences between groups. Furthermore, analysis of the academic year variable was performed using one-way ANOVA. The last steps of analysis in this study, used multivariate analysis.

## 4 Results

### 4.1 Respondent characteristics

Based on Table 1, the mean age of the 470 medical students was 20.7 years (SD = 2.4), ranging from 17 to 26 years. The majority of respondents were female (*n* = 316, 67.2%). Participants were distributed across academic levels, with the largest proportion in the first year (25.1%) and clinical rotation stage (24.7%).

**TABLE 1:** Respondent characteristics.

Characteristics	N (470)	%
Age		
17	13	2.8%
18	55	11.7%
19	107	22.8%
20	85	18.1%
21	55	11.7%
22	38	8.1%
23	24	5.1%
24	70	14.9%
25	21	4.5%
26	2	0.4%
Total	470	100%
Gender		
Male	154	32.8%
Female	316	67.2%
Total	470	100%
Academic year		
First year	118	25.1%
Second year	101	21.5%
Third year	71	15.1%
Fourth year	64	13.6%
Clinical rotation stage	116	24.7%
Total	470	100%

### 4.2 Medical students’ perceptions of spiritual care based on respondents’ demographic characteristics

Based on Table 2, the overall mean spiritual care perception score was 76.11 (SD = 7.02), indicating generally positive perceptions among students. Female students demonstrated slightly higher scores than males. Across academic levels, scores increased progressively, with clinical-stage students reporting the highest mean values.

**TABLE 2:** Minimum, maximum, mean, and standard deviation of medical students’ perceptions of spiritual care based on demographic characteristics.

Demographic characteristics	n	Min	Max	Mean	SD
Gender					
Male	154	54.00	88.00	75.12	6.93
Female	316	54.00	90.00	76.59	7.02
Academic year					
First year	118	54.00	90.00	74.05	7.16
Second year	101	54.00	90.00	76.30	6.66
Third year	71	54.00	90.00	76.65	7.37
Fourth year	64	62.00	89.00	76.75	5.95
Clinical rotation stage	116	61.00	90.00	77.36	7.17
Total medical student respondents	470	54.00	90.00	76.11	7.02

### 4.3 Relationship between demographic characteristics and perceptions of spiritual care

Based on the one-way ANOVA test in the Table 3, a significance value (sig.) of 0.005 (sig. < 0.05) was obtained, indicating a relationship between academic year and perceptions of spiritual care. The lowest mean score of spiritual care perception was observed among first-year students, whereas the highest mean score was found among students in the clinical rotation stage. In addition, the findings showed that higher academic years were associated with higher spiritual care perception scores. To identify differences between groups, a *post hoc* Bonferroni test was subsequently performed.

**TABLE 3:** Bivariate analysis of the relationship between demographic characteristics and perceptions of spiritual care.

Demographic characteristics		n	Mean ± SD spiritual care	*p*-value	*Post hoc* Bonferroni
Academic year	First year (a)	118	74.05 ± 7.16	0.005*	a-b: 0.173
	Second year (b)	101	76.30 ± 6.66		a-c: 0.130
	Third year (c)	71	76.65 ± 7.37		a-d: 0.125
	Fourth year (d)	64	76.75 ± 5.95		**a-e: 0.003**
	Clinical rotation stage (e)	116	77.36 ± 7.17		b-c: 1.000
					b-d: 1.000
					b-e: 1.000
					c-d: 1.000
					c-e: 1.000
					d-e: 1.000
Gender	Male	154	75.12 ± 6.93	0.032**	NA
	Female	316	76.59 ± 7.02
Age				0.001*** **(*r* = 0.153)**	NA

*One-way ANOVA. **Independent *t*-test. ***Pearson correlation test. Bold values indicate statistically significant results (*p* < 0.05).

Based on the *post hoc* Bonferroni test, the data were considered significant when the significance value was less than 0.05 (sig. < 0.05), indicating differences between groups. The significant difference was observed between first-year students and students in the clinical rotation stage, with a significance value of 0.003 (sig. < 0.05).

Based on the independent *t*-test, female students were found to have slightly higher spiritual care perception scores than male students. The significance value obtained was 0.032 (sig. < 0.05), indicating that gender influenced perceptions of spiritual care.

Based on Pearson’s correlation analysis between age and perceptions of spiritual care, a significance value of 0.001 (sig. < 0.05) and a correlation coefficient of 0.153 were obtained. This finding indicates a significant positive relationship between age and perceptions of spiritual care, suggesting that higher age is associated with better perceptions of spiritual care. However, the strength of this relationship was very weak, as the r value was below 0.2 (*r* < 0.2).

Based on Table 4, in Domain 1 (Existential Element), the data showed that age, gender, and academic year did not have a significant effect on spiritual care perception scores in Domain 1, as all significance values were greater than 0.05 (sig. > 0.05). In Domain 2 (Religiosity), the data indicated that age and academic year did not have a significant effect on spiritual care perception scores in Domain 2, as their significance values were greater than 0.05 (sig. > 0.05). In contrast, gender had a significant effect on spiritual care perception scores in Domain 2, as the significance value was less than 0.05 (sig. < 0.05). Based on the independent *t*-test, female students had slightly higher Domain 2 spiritual care perception scores than male students.

**TABLE 4:** Bivariate analysis of the relationship between demographic characteristics and perceptions of spiritual care by domain.

Demographic characteristics		Domain 1 (existential element)	Domain 2 (religiosity)	Domain 3 (personalized care)	Domain 4 (spiritual care)	Domain 5 (Muslim’s value)
Age						
*p-*value* (r)		0.546 (0.028)	0.090 (−0.078)	**0.000** **(0.229)**	**0.000** **(0.235)**	**0.000** **(0.270)**
Gender						
*p-*value**		0.109	**0.025**	0.072	0.334	**0.023**
Male	**Mean ± SD**	21.58 ± 2.52	12.29 ± 1.78	11.23 ± 1.64	25.63 ± 2.79	4.25 ± 0.83
Female	**Mean ± SD**	21.97 ± 2.51	12.67 ± 1.69	11.53 ± 1.82	25.89 ± 2.74	4.42 ± 0.67
Academic year						
*p*-value***		0.311	0.058	**0.000**	**0.000**	**0.000**
First year (a)	**Mean ± SD**	21.52 ± 2.39	12.61 ± 1.74	10.95 ± 1.55	24.69 ± 2.98	4.13 ± 0.81
Second year (b)	**Mean ± SD**	21.98 ± 2.46	12.55 ± 1.74	11.25 ± 1.84	26.00 ± 2.64	4.27 ± 0.85
Third year (c)	**Mean ± SD**	22.20 ± 2.55	12.48 ± 1.63	11.32 ± 1.65	26.07 ± 2.84	4.41 ± 0.65
Fourth year (d)	**Mean ± SD**	22.11 ± 2.19	13.03 ± 1.48	11.50 ± 1.57	25.50 ± 2.22	4.36 ± 0.68
Clinical rotation stage (e)	**Mean ± SD**	21.70 ± 2.81	12.23 ± 1.86	12.13 ± 1.90	26.78 ± 2.45	4.67 ± 0.49
				***Post-hoc*** **Bonferroni**	***Post-hoc*** **Bonferroni**	***Post-hoc*** **Bonferroni**
				a-b: 1.000	**a-b: 0.003**	a-b: 1.000
				a-c: 1.000	**a-c: 0.006**	a-c: 0.086
				a-d: 0.399	a-d: 0.497	a-d: 0.356
				**a-e: 0.000**	**a-e: 0.000**	**a-e: 0.000**
				b-c: 1.000	b-c: 1.000	b-c: 1.000
				b-d: 1.000	b-d: 1.000	b-d: 1.000
				**b-e: 0.002**	b-e: 0.309	**b-e: 0.000**
				c-d: 1.000	c-d: 1.000	c-d: 1.000
				**c-e: 0.020**	c-e: 0.758	c-e: 0.140
				d-e: 0.194	**d-e: 0.021**	**d-e: 0.048**

*One Way Anova Test. **The independent *t*-test. ***Pearson Correlation. Bold values indicate statistically significant results (*p* < 0.05).

In Domain 3 (Personalized Care), the data from Table 4 showed that gender did not have a significant effect on spiritual care perception scores in Domain 3, as the significance value was greater than 0.05 (sig. > 0.05). In contrast, age and academic year had a significant effect on spiritual care perception scores in Domain 3, as their significance values were less than 0.05 (sig. < 0.05). Pearson’s correlation analysis yielded a correlation coefficient of 0.229, indicating a significant positive relationship between age and Domain 3 spiritual care perception scores, such that higher age was associated with better perceptions of spiritual care in Domain 3. However, the strength of this relationship remained very weak, as the correlation coefficient fell within the range of 0.2–0.4. Based on the *post hoc* Bonferroni test, significant differences were observed between first-year students and those in the clinical rotation stage, second-year students and those in the clinical rotation stage, and third-year students and those in the clinical rotation stage (sig. < 0.05).

In Domain 4 (Spiritual Care) from Table 4, the data indicated that gender did not have a significant effect on spiritual care perception scores in Domain 4, as the significance value was greater than 0.05 (sig. > 0.05). In contrast, age and academic year had a significant effect on spiritual care perception scores in Domain 4, as their significance values were less than 0.05 (sig. < 0.05). Pearson’s correlation analysis yielded a correlation coefficient of 0.235, indicating a significant positive relationship between age and Domain 4 spiritual care perception scores, such that higher age was associated with better perceptions of spiritual care in Domain 4. However, the strength of this relationship remained very weak, as the correlation coefficient fell within the range of 0.2–0.4. Based on the *post hoc* Bonferroni test, significant differences were observed between first-year and second-year students, first-year and third-year students, first-year students and those in the clinical rotation stage, and fourth-year students and those in the clinical rotation stage (sig. < 0.05).

In Domain 5 (Muslim’s value) from Table 4, the data showed that age, gender, and academic year had a significant effect on spiritual care perception scores in Domain 5, as all significance values were less than 0.05 (sig. < 0.05). Pearson’s correlation analysis yielded a correlation coefficient of 0.270, indicating a significant positive relationship between age and Domain 5 spiritual care perception scores, such that higher age was associated with better perceptions of spiritual care in Domain 5. However, the strength of this relationship remained very weak, as the correlation coefficient fell within the range of 0.2–0.4. Based on the independent *t*-test, female students had slightly higher Domain 5 spiritual care perception scores than male students. Based on the *post hoc* Bonferroni test, significant differences were observed between first-year and second-year students, first-year and third-year students, first-year students and those in the clinical rotation stage, and fourth-year students and those in the clinical rotation stage (sig. < 0.05).

Based on the data in the Table 5, the total spiritual care score showed that age and academic year did not have a significant effect on the total spiritual care perception score, as their significance values were greater than 0.05 (sig. > 0.05). In contrast, gender had a significant effect on the total spiritual care perception score, as the significance value was less than 0.05 (sig. < 0.05). The adjusted R-squared value obtained was 3%, indicating that the independent variables—age, gender, and academic year—accounted for 3% of the variance in the total spiritual care perception score. The resulting regression equation was *Y* = 62.906 + 1.460 (Gender).

**TABLE 5:** Multivariate analysis results.

Demographic characteristics	Spiritual care (total score)	1st domain (existential element)	2nd domain (religiosity)	3rd domain (personalized care)	4th domain (spiritual care)	5th domain (Muslim’s value)
Age	0.247	0.133	0.474	0.834	0.182	0.124
Gender	**0.035**	0.063	**0.027**	0.200	0.358	**0.026**
Male						
Female						
Academic year						
Second year	0.059	0.428	0.900	0.245	**0.003**	0.375
Third year	0.170	0.547	0.914	0.282	**0.047**	0.205
Fourth year	0.457	0.869	0.134	0.241	0.769	0.848
Clinical rotation stage	0.808	0.219	0.982	0.084	0.322	0.470
Adjusted R^2^	3%	0.9%	1.9%	5.1%	7%	7.6%
Linear regression equations	*Y* = 62.906 + 1.460 (Gender)	–	*Y* = 13.317 + 0.379 (Gender)	–	*Y* = 20.409 + 1.138 second year + 0.997 third year	*Y* = 2.680 + 0.157 Gender

Bold values indicate statistically significant results (*p* < 0.05).

In Domain 1 (Existential Element) as stated in the Table 5, the data showed that age, gender, and academic year did not have a significant effect on spiritual care perception scores in Domain 1, as all significance values were greater than 0.05 (sig. > 0.05). In Domain 2 (Religiosity), the data showed that age and academic year did not have a significant effect on spiritual care perception scores in Domain 2, as their significance values were greater than 0.05 (sig. > 0.05). In contrast, gender had a significant effect on spiritual care perception scores in Domain 2, as the significance value was less than 0.05 (sig. < 0.05). The adjusted R-squared value obtained was 1.9%, indicating that the independent variables—age, gender, and academic year—accounted for 1.9% of the variance in Domain 2 (Religiosity). The resulting regression equation was *Y* = 13.317 + 0.379 (Gender).

In Domain 3 (Personalized Care) from Table 5, the data showed that age, gender, and academic year did not have a significant effect on spiritual care perception scores in Domain 3, as all significance values were greater than 0.05 (sig. > 0.05). In Domain 4 (Spiritual Care), the data showed that age and gender did not have a significant effect on spiritual care perception scores in Domain 4, as their significance values were greater than 0.05 (sig. > 0.05). In contrast, second-year and third-year academic status had a significant effect on spiritual care perception scores in Domain 4, as their significance values were less than 0.05 (sig. < 0.05). The adjusted R-squared value obtained was 7%, indicating that the independent variables—age, gender, and academic year—accounted for 7% of the variance in Domain 4 (Spiritual Care). The resulting regression equation was Y = 20.409 + 1.138 (second year) + 0.997 (third year).

Based on Table 5, in domain 5 (Muslim’s Value), the data showed that age and academic year did not have a significant effect on spiritual care perception scores in Domain 5, as their significance values were greater than 0.05 (sig. > 0.05). In contrast, gender had a significant effect on spiritual care perception scores in Domain 5, as the significance value was less than 0.05 (sig. < 0.05). The adjusted R-squared value obtained was 7.6%, indicating that the independent variables—age, gender, and academic year—accounted for 7.6% of the variance in Domain 5 (Muslim’s Value). The resulting regression equation was *Y* = 2.680 + 0.157 (Gender).

## 5 Discussion

Based on data from all 470 medical student respondents, the lowest spiritual care perception score was 54.00 and the highest score was 90.00, with an overall mean of 76.11 and a standard deviation of 7.02. These findings are consistent with the study by Aksoy and Coban (18), which was conducted among first-, second-, third-, and fourth-year students of the Faculty of Health Sciences at Ataturk University to examine perceptions of spiritual care. That study reported that students had good perceptions of spirituality and spiritual care. In line with these findings, it can be stated that the students included in the present study demonstrated good perceptions of spirituality and spiritual care.

Another study by Atkinson et al. (19), involving 165 medical students undertaking clerkships in surgical and anesthesia rotations, found that respondents showed a strong interest in providing spiritual care to patients. Furthermore, the majority of respondents (72%) stated that meeting patients’ spiritual needs would greatly influence patient treatment outcomes.

Based on the findings of this study, which included three factors—age, gender, and academic year—all three factors were statistically associated with the total spiritual care perception score. However, after multivariate linear regression analysis was performed, only gender remained a significant predictor of the total spiritual care perception score.

With regard to age, a positive relationship was found between age and perceptions of spiritual care, indicating that higher age was associated with better perceptions of spiritual care. This finding is consistent with the study by Babamohamadi et al. (20), which reported a significant relationship between age and spiritual care perceptions among students. Similarly, Kaddourah et al. (21) found that older respondents tended to have slightly better perceptions of spiritual care than younger respondents. This may be attributed to the fact that older respondents have more experience, which enables them to provide spiritual care more effectively than younger individuals (21). However, in the present study, age did not have a significant effect on perceptions of spiritual care after multivariate analysis. This finding is in line with the study by Melhem et al. (15), which reported that age was not associated with perceptions of spiritual care, indicating that perceptions of spiritual care did not necessarily improve with increasing age. This discrepancy in findings may be due to other demographic characteristics, such as race, ethnicity, and religion, which were not examined in this study, as well as differences in the number of respondents across age groups.

With regard to gender, the findings showed that female students had slightly higher spiritual care perception scores than male students, and the significance value was less than 0.05, indicating that gender influenced perceptions of spiritual care. This finding may be explained by the notion that women are more capable of sharing feelings and emotions than men (20). In line with this, Kalkim et al. (22) also reported that women had better perceptions of spiritual care than men. This result is consistent with the study by Melhem et al. (15), which found that gender had a significant effect on perceptions of spiritual care. That study suggested that, in patient care, women tend to focus more on emotional and affective aspects, whereas men tend to focus more on physical aspects.

In this study, a relationship was found between academic year and perceptions of spiritual care. The lowest mean spiritual care perception score was observed among first-year students, whereas the highest mean score was found among students in the clinical rotation stage. In addition, the findings indicated that higher academic years were associated with higher spiritual care perception scores. This finding is consistent with the study by Aslan and Unsal (23), which reported a significant relationship between year of study and perceptions of spiritual care. This may be attributed to the fact that students in the clinical rotation stage have received more academic instruction and have greater experience in patient care compared with students in the first, second, third, and fourth academic years. Students who have received education related to spirituality and spiritual care tend to obtain higher spirituality and spiritual care perception scores (24).

The present study also showed that the comparison between first-year students and those in the clinical rotation stage yielded the smallest significance value among all group pairs. However, after multivariate analysis was conducted, academic year did not have a significant effect on perceptions of spiritual care. Similarly, Aksoy and Coban (18) reported that academic year did not influence perceptions of spiritual care. These differences in findings may be due to variations in the number of respondents across academic year groups, which can affect the statistical significance of the results.

Beyond overall scores, this study also provides insight into domain-specific perceptions of spiritual care. The findings showed that Domains related to personalized care, spiritual care, and Muslim values were significantly associated with age and academic level. This suggests that experiential learning and increased clinical exposure may enhance students’ ability to understand and apply spiritual care in practice.

The higher scores observed in the “Muslim’s Value” domain reflect the strong influence of religious and cultural context in shaping students’ perceptions. This is consistent with previous studies indicating that spirituality in healthcare is deeply embedded within cultural and religious frameworks (25).

This study found that medical students generally have positive perceptions of spiritual care. Among the examined variables, gender emerged as the only significant predictor in multivariate analysis, with female students demonstrating higher perception scores.

Although age and academic year showed significant associations in bivariate analysis, their effects were not sustained in multivariate analysis. This suggests that their influence may be indirect or influenced by other factors not measured in this study.

According to the study by Quigley et al. (26), several factors contribute to the suboptimal provision of spiritual care by healthcare professionals, including limited knowledge and perceptions of spiritual care. This condition may result from the lack of clear guidelines for healthcare professionals, time constraints, limited self-confidence, as well as insufficient training and education related to spiritual care. Education on spirituality and spiritual care is therefore important to be developed within educational curricula to broaden knowledge and enhance competence in providing spiritual care to patients (26).

## 6 Study limitations

The generalizability of the findings may be limited, as the study was conducted within a single university in Indonesia and involved participants from one medical faculty. However, the comprehensiveness of the analyses and the multiple characteristics (academic years and clinical stages) of the respondents within large sample size in this study provide a basis for further use of this result. Furthermore, this study employed a self-report questionnaire, which has inherent limitations due to potential bias. However, the comprehensiveness of the analyses of the study provide a basis for further validation and use of this study. As a practical recommendation, future researchers using mixed methods for data collection are advised as further validation studies. This approach may encourage a greater sense of appreciation among participants, thereby increasing their commitment when completing the questionnaire.

## 7 Conclusion

Based on this study, medical students demonstrated generally positive perceptions of spiritual care, with variations observed across demographic characteristics. Gender was identified as a significant factor, with female students reporting higher perception scores, while academic level showed an association in bivariate analysis, with students in more advanced stages tending to report higher scores.

These findings contribute to a better understanding of factors associated with medical students’ perceptions of spiritual care, particularly within a Muslim-context educational setting. However, given the cross-sectional nature of the study, no causal inferences can be made regarding the development of perceptions or the impact of educational exposure.

Future studies employing longitudinal or experimental designs are recommended to examine how educational interventions may influence both perceptions and competencies related to spiritual care. Additionally, multicenter studies could provide a broader perspective on how cultural and institutional factors shape these perceptions.

## Data Availability

The original contributions presented in this study are included in the article/supplementary material, further inquiries can be directed to the corresponding author.
